# Recent advances in epigenetic therapeutics for Rett syndrome: from mechanisms to clinical trials

**DOI:** 10.3389/fnbeh.2026.1835425

**Published:** 2026-06-30

**Authors:** Cuijie Zhao, Zhuo Huang, Ruixing Li, Lei Hua, Huawei Li, Bocai Wang

**Affiliations:** 1 Department of Pediatrics, The First Affiliated Hospital, Henan University of Chinese Medicine, Zhengzhou, Henan, China; 2 School of Pediatrics, Henan University of Chinese Medicine, Zhengzhou, Henan, China

**Keywords:** epigenetic editing, gene therapy, MeCP2, precision medicine, Rett syndrome

## Abstract

Rett syndrome (RTT) stands at the forefront of the genetic therapy revolution. This severe X-linked neurodevelopmental disorder, primarily caused by mutations in the *MECP2* gene, was historically considered a static condition but is now recognized as a potentially reversible neurodevelopmental disorder. This review synthesizes recent breakthroughs in our understanding of MeCP2’s role in chromatin architecture, including its involvement in liquid-liquid phase separation (LLPS). We critically examine the transition from conventional symptom management to precision epigenetic therapeutics. Key advances discussed include next-generation gene replacement strategies with autoregulatory control to prevent toxicity, programmable epigenetic editing (e.g., CRISPR-off/on) to correct *MECP2* expression endogenously, and novel approaches for X-chromosome reactivation (XCI). Furthermore, we propose a stratified therapeutic framework (genotype-guided therapies) based on specific mutation types. Finally, we analyze data from ongoing clinical trials and highlight the remaining hurdles—such as delivery efficiency, immunogenicity, and the urgent need for objective biomarkers—that must be overcome to translate these epigenetic innovations into a cure.

## 1 Introduction

### 1.1 The clinical burden of Rett syndrome: beyond a rare disease

Rett syndrome (RTT) represents a devastating chapter in pediatric neurology, standing as the second most common cause of severe intellectual disability in females after Down syndrome, with an estimated prevalence of 1 in 10,000 to 15,000 live female births (Percy et al., 2024). The clinical trajectory is heartbreakingly distinct: following a period of apparently normal development for the first 6–18 months, children enter a “regression phase,” losing acquired purposeful hand skills and spoken language. This is followed by the onset of stereotypic hand movements (e.g., wringing, washing), gait abnormalities, and a spectrum of comorbidities including refractory epilepsy, autonomic dysfunction, and severe scoliosis (Ferreira et al., 2025). For decades, management was strictly supportive, focusing on symptom mitigation rather than disease modification.

### 1.2 The genetic basis: MECP2 as an epigenetic “Reader”

In 1999, the discovery that loss-of-function mutations in the MECP2 (methyl-CpG-binding protein 2) gene on the X chromosome (Xq28) cause RTT fundamentally shifted our understanding of the disease (Amir et al., 1999). Unlike metabolic enzymopathies or structural protein defects, RTT is a disorder of “epigenetic interpretation.” MeCP2 is a ubiquitous nuclear protein that binds to methylated cytosines (5mC) and recruits co-repressor complexes to dampen transcriptional noise (Lyst and Bird, 2015). Although MeCP2 is expressed in almost all somatic cells, its absence in peripheral tissues produces remarkably mild physiological consequences. In stark contrast, the central nervous system is acutely vulnerable to its loss because mature neurons demand exceptionally high levels of MeCP2 for complex gene regulation (Chen et al., 2001). Consequently, Its deficiency does not kill neurons but rather disrupts the precise “fine-tuning” of gene expression required for synaptic maturation and plasticity (Chahrour and Zoghbi, 2007).

### 1.3 The paradigm shift: reversibility and the dawn of molecular medicine

The dogma that neurodevelopmental disorders are irreversible was shattered in 2007 by a landmark study in science. Guy et al. (2007) demonstrated that re-expressing *Mecp2* in adult symptomatic mice resulted in a robust reversal of neurological defects, proving that the RTT brain is not structurally atrophied but functionally dormant. This proof-of-concept has since been substantiated in patient-derived brain organoids (Samarasinghe et al., 2021), demonstrating that restoring MeCP2 signaling can rescue electrophysiological homeostasis even in fully differentiated mature neuronal networks. Additionally, recent studies have confirmed the safety and feasibility of gene therapy in non-human primate models (Powers et al., 2023). This established RTT as a condition of synaptic failure rather than neurodegeneration, igniting a race to develop curative therapies.

### 1.4 Scope of review: bridging the gap between epigenetic mechanisms and clinical trials

Today, we stand at a historic inflection point. The FDA approval of Trofinetide in 2023 marked the first available treatment for RTT, yet it remains a symptomatic therapy (Neul et al., 2023). The field is now pivoting toward disease-modifying strategies that target the epigenetic root cause. This review critically examines the latest advances in epigenetic therapeutics, from next-generation gene replacement strategies (e.g., TSHA-102) (Sadhu et al., 2023) to cutting-edge epigenetic editing (CRISPR-based X-reactivation) (Qian et al., 2023). We aim to provide a roadmap for understanding how deep mechanistic insights into MeCP2 biology are translating into precision medicines that could one day rewrite the natural history of this condition.

## 2 MeCP2: the master epigenetic regulator (an updated view)

### 2.1 The classical view vs. new insights: repressor, activator, or architect?

Historically, MeCP2 was categorized strictly as a transcriptional repressor that binds to methylated CpG dinucleotides. However, this “one-dimensional” view fails to explain the pervasive alterations in chromatin structure observed in RTT. Emerging evidence suggests that MeCP2 functions primarily as a global chromatin architect (Skene et al., 2010). Rather than silencing specific target genes individually, it dampens transcriptional noise genome-wide by bridging nucleosomal DNA and organizing higher-order chromatin topology (Skene et al., 2010). Its loss leads not to a simple “upregulation” of targets, but to a systemic destabilization of the neuronal epigenome.

### 2.2 Binding specificity: reading methylation (5mC) and hydroxymethylation (5hmC)

A critical update in MeCP2 biology is its specific affinity for non-canonical epigenetic marks. While it binds 5-methylcytosine (5mC) in the classic CpG context, MeCP2 is also the primary reader of methylation in the non-CpG context (mCH, where H = A, T, or C). Crucially, mCH accumulates in neurons postnatally, coinciding precisely with the onset of RTT symptoms (6–18 months), suggesting that the failure to read this “maturation signal” is the disease driver (Chen et al., 2015). Furthermore, MeCP2 also interacts with 5-hydroxymethylcytosine (5hmC) (Mellén et al., 2012), and recent thermodynamic studies (2023) have elucidated its binding dynamics (Chua et al., 2023), implying a dual role in both repression and activation depending on the epigenetic context.

### 2.3 Chromatin compaction and liquid-liquid phase separation (LLPS)

How does one protein organize an entire genome? The answer lies in physics. MeCP2 contains extensive intrinsically disordered regions (IDRs) which allow it to undergo liquid-liquid phase separation (LLPS). Through this mechanism, MeCP2 molecules condense into liquid droplets, pulling heterochromatin into compact, silent domains (Li et al., 2020). Seminal research (2020) proposed that this phase separation capability is essential for heterochromatin assembly and that many RTT-causing mutations disrupt the protein’s ability to form these repressive condensates, leading to “leaky” heterochromatin (Wang et al., 2020). Crucially, the degree of LLPS disruption often correlates with disease severity; mutations that severely impair condensate formation tend to result in more profound chromatin disorganization and clinical deficits. This finding opens a new therapeutic frontier: identifying small molecules capable of stabilizing MeCP2 condensates or restoring the phase-separation properties of mutant proteins.

### 2.4 The MeCP2-NCoR/SMRT co-repressor complex: the molecular brake

**FIGURE 1:**
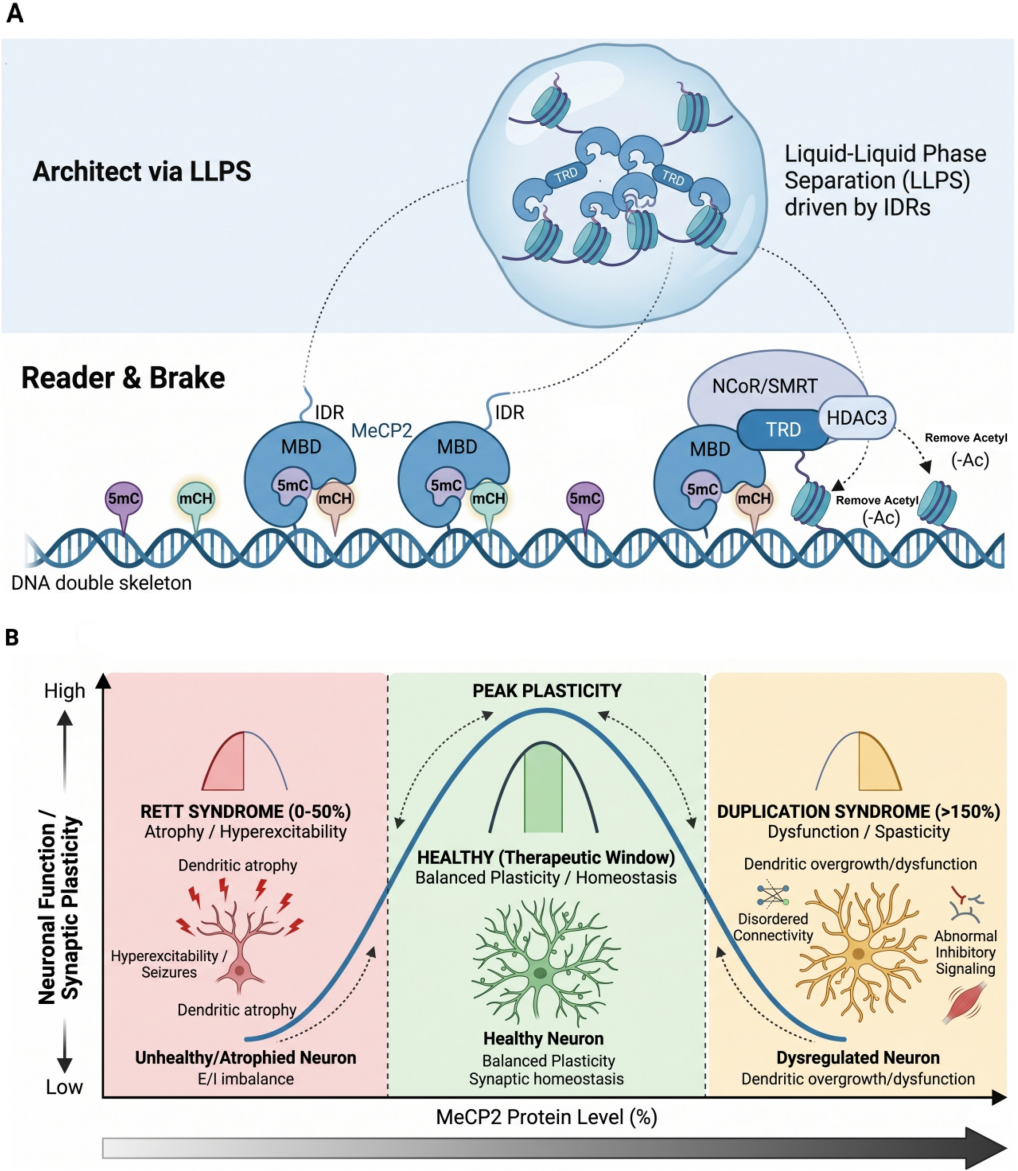
The multifaceted role of MeCP2: from chromatin architecture to the “Goldilocks” effect. **(A)** Molecular mechanisms of MeCP2 in transcriptional regulation and chromatin architecture. **(B)** The “Goldilocks” effect of MeCP2 dosage on neuronal function and synaptic plasticity.

While LLPS provides the structural compaction, the functional silencing is achieved through the recruitment of the NCoR/SMRT co-repressor complex. NCoR (nuclear receptor corepressor) and SMRT (silencing mediator for retinoid and thyroid hormone receptors) are scaffolding proteins that assemble a large multi-subunit complex responsible for turning off gene transcription (Mottis et al., 2013). MeCP2 acts as a bridge, recruiting this complex—which prominently features histone deacetylase 3 (HDAC3) to methylated DNA loci (Nott et al., 2016). This deacetylation of histone tails (e.g., H3K27ac removal) “locks” the chromatin in a closed state. Disrupting the interaction between MeCP2 and the NCoR/SMRT complex causes a RTT-like phenotype even if MeCP2 can still bind DNA, highlighting that “reading” (DNA binding) and “acting” (co-repressor recruitment) are two distinct but equally vital functions (Lyst et al., 2013). The diverse molecular functions of MeCP2 (LLPS, transcriptional braking, co-repressor recruitment) and the critical dosage-dependent ‘Goldilocks’ effect are presented (Figure 1).

## 3 Pathophysiological consequences of epigenetic dysregulation

### 3.1 Transcriptional noise and long gene misregulation

The loss of MeCP2’s repressive function does not result in chaotic gene expression, but rather a subtle, widespread amplification of “transcriptional noise.” A distinct feature of RTT pathology is the preferential upregulation of long genes (> 100 kb) and repetitive elements (e.g., L1 retrotransposons) which are normally heavily methylated and silenced in neurons (Moore et al., 2024). Mechanistically, long genes accumulate exceptionally high levels of non-CG methylation (mCH) across their vast transcribed regions. MeCP2 coats these extended gene bodies by binding to mCH, acting as a “transcriptional damper” or “speed bump” that restricts the elongation rate of RNA Polymerase II (Pol II) (Gabel et al., 2015). This “length-dependent” misregulation suggests that MeCP2 acts as a processivity factor, ensuring controlled transcription and preventing the transcriptional machinery from stalling or initiating spuriously at cryptic promoters within these long genomic stretches. The reactivation of retrotransposons may further contribute to genomic instability in RTT neurons (Muotri et al., 2010).

### 3.2 Synaptic homeostasis and E/I imbalance (the glutamate/GABA switch)

The clinical hallmark of RTT—seizures—stems from a fundamental disruption in the Excitatory/Inhibitory (E/I) balance (Dani et al., 2005). MeCP2 is critical for regulating the expression of Gad1 and Gad2, the enzymes responsible for GABA synthesis (Chao et al., 2010). Mechanistically, MeCP2 binds to the regulatory regions of these genes and recruits transcriptional co-activators, such as CREB, to maintain an open, active chromatin state (Chahrour et al., 2008). In MeCP2-deficient brains, the loss of this transactivation leads to reduced transcription of Gad1 and Gad2; consequently, GABAergic signaling is dampened while glutamatergic transmission remains unchecked, leading to hyperexcitability (Calfa et al., 2015). This synaptic instability is not merely a wiring defect but a functional failure of homeostatic plasticity, rendering neural circuits unable to adjust their gain in response to input (Blackman et al., 2012).

### 3.3 Downstream targets: BDNF and the neurotrophic hypothesis

Among the thousands of dysregulated genes, Brain-Derived Neurotrophic Factor (BDNF) stands out as the most critical downstream effector. MeCP2 normally regulates BDNF expression in an activity-dependent manner (Chen et al., 2003). Mechanistically, in healthy neurons, membrane depolarization and calcium influx trigger the phosphorylation of MeCP2 (e.g., at Serine 421), causing its dissociation from the Bdnf promoter to permit robust transcription (Zhou et al., 2006). In RTT, this dynamic transactivation is uncoupled; mutant MeCP2 fails to respond to these physiological signals, leaving the Bdnf gene locked in a repressed state. In RTT, BDNF levels are consequently chronically low, leading to dendritic atrophy and reduced synaptic connectivity (Larimore et al., 2009). Restoring BDNF signaling has been shown to rescue multiple phenotypes in mouse models, validating it as a key therapeutic target (Chang et al., 2006). To achieve this, researchers have explored direct gene delivery (e.g., AAV-BDNF), small-molecule TrkB receptor agonists (e.g., LM22A-4) to mimic BDNF binding, and pharmacological modulators (like sub-anesthetic ketamine) to stimulate endogenous production (Schmid et al., 2012; Kron et al., 2012). Although promising, severe delivery challenges remain; native BDNF protein exhibits poor blood-brain barrier (BBB) permeability and a short serum half-life, while systemic administration triggers profound peripheral toxicity (such as hyperalgesia) due to off-target TrkB activation in the peripheral nervous system (Miranda-Lourenço et al., 2020).

### 3.4 Non-neuronal contributors: the role of glia in epigenetic support

RTT is not solely a “neuron-autonomous” disease. MeCP2-deficient astrocytes and microglia fail to support neuronal health. Astrocytes exhibit impaired glutamate clearance, exacerbating excitotoxicity (Okabe et al., 2012), while microglia release excessive pro-inflammatory cytokines, creating a neurotoxic environment (Derecki et al., 2012). Re-expressing MeCP2 specifically in glial cells can arrest disease progression in mice, highlighting the need for therapeutics to target the entire neuro-glial unit, not just neurons (Lioy et al., 2011).

### 3.5 Genotype-phenotype correlations: from mutation type to therapeutic strategy

Understanding the specific *MECP2* mutation is no longer just for diagnosis; it dictates therapeutic strategy.

**FIGURE 2:**
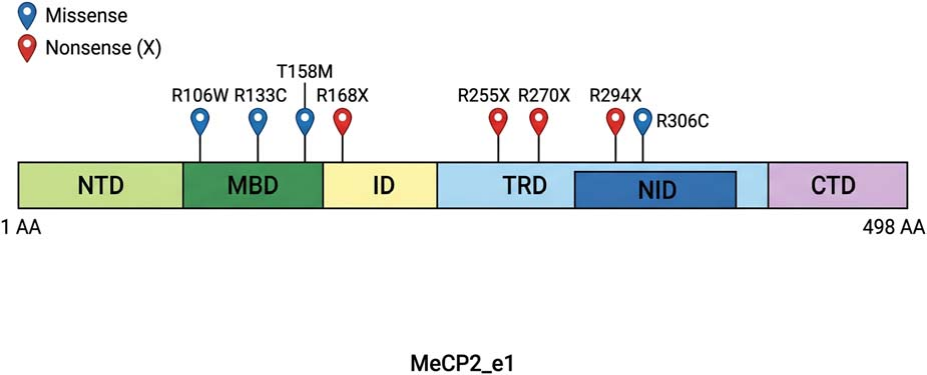
Schematic architecture of the *MeCP2* protein illustrating functional domains and the localization of the eight most prevalent hotspot mutations in Rett syndrome.

While over 900 pathogenic variants have been identified, eight “hotspot” mutations (R106W, R133C, T158M, R168X, R255X, R270X, R294X, and R306C) account for over 60% of all classic RTT cases (Cuddapah et al., 2014). The specific domain location of these mutations profoundly dictates the functional consequence on the MeCP2 protein and correlates strongly with clinical severity (summarized in Table 1 and illustrated in Figure 2).

**TABLE 1:** Summary of hotspot *MECP2* mutations: prevalence, functional consequences, and clinical severity.

Mutation	Type	Domain	Est. prevalence (%)*	Functional consequence on MeCP2 protein	Typical clinical severity
T158M	Missense	MBD	9.0%–12.0%	Destabilizes protein structure; critically reduces binding to methylated DNA.	Severe
R168X	Non-sense	TRD	9.0%–11.0%	Early truncation; complete loss of NCoR/SMRT interaction domain (NID).	Severe
R255X	Non-sense	TRD	5.0%–9.0%	Truncation before NID; fails to recruit co-repressor complex.	Severe
R270X	Non-sense	TRD	5.0%–9.0%	Truncation before NID; fails to recruit co-repressor complex.	Severe
R294X	Non-sense	TRD	5.0%–8.0%	Late truncation (after NID); partially retains NCoR/SMRT binding capacity.	Mild
R306C	Missense	TRD (NID)	5.0%–7.0%	Abolishes interaction with NCoR/SMRT complex, but retains DNA binding.	Mild to Moderate
R133C	Missense	MBD	4.0%–5.0%	Retains partial DNA binding (especially to 5hmC); forms functional condensates.	Mild
R106W	Missense	MBD	3.0%–4.0%	Completely abolishes DNA binding capacity.	Sever

*Estimated prevalence data are derived from large cohort natural history studies (Neul et al., 2008; Cuddapah et al., 2014). MBD, methyl-binding domain; TRD, transcriptional repression domain; NID, NCoR/SMRT interaction domain.

Missense mutations, such as R133C and T158M, illustrate the need for tailored approaches. While R133C occurs in the methyl-binding domain (MBD) but retains partial binding capacity—leading to a milder phenotype—T158M destabilizes the protein structure and drives severe disease; thus, protein stabilizers or chaperones represent a promising avenue for T158M carriers (Lombardi et al., 2017). Similarly, C-terminal truncations (e.g., R294X) often result in proteins retaining MBD and NCoR binding domains, correlating with milder phenotypes and preserved ambulation (Neul et al., 2008). For these individuals, “fine-tuning” therapies that enhance the residual protein’s stability or function may prove safer and sufficient compared to full gene replacement (Collins et al., 2022). In contrast, nonsense mutations (e.g., R168X, R255X), which account for approximately 35% of cases, lead to truncated, non-functional proteins. These patients are ideal candidates for read-through therapy or RNA editing to repair the premature stop codon (Merritt et al., 2020; Sinnamon et al., 2020). However, despite theoretical promise, first-generation read-through agents like gentamicin are limited by toxicity and poor blood-brain barrier penetrance, necessitating the development of safer, non-aminoglycoside alternatives (Vecsler et al., 2011; Li et al., 2026). This stratification represents the first step toward personalized medicine in RTT.

## 4 Therapeutic strategy I: gene replacement therapy 2.0

### 4.1 The “Goldilocks” challenge: defining the therapeutic window of MeCP2

Gene replacement therapy for RTT faces a unique “Goldilocks” challenge: MECP2 expression must be “just right.” While too little MeCP2 causes RTT, too much causes MECP2 Duplication Syndrome, a severe condition characterized by spasticity and epilepsy (Shao et al., 2021; Pehlivan et al., 2024). This narrow therapeutic window doomed first-generation vectors, which used strong, unregulated promoters (like CMV) that risked toxic overexpression in transduced cells. The field realized that simple “replacement” is dangerous; “regulated restoration” is required.

### 4.2 First-generation approaches and toxicity concerns

Early preclinical studies using AAV9 vectors demonstrated that while they extended survival in RTT mice, they often caused liver toxicity and neurological deficits in wild-type control mice (mimicking overexpression) (Sinnett et al., 2017; Matagne et al., 2021). This highlighted a critical safety hurdle for human trials: since female patients are mosaics (having a mix of MeCP2-null and MeCP2-normal cells due to random X-inactivation), delivering a gene therapy vector would inevitably “double dose” the healthy cells, potentially triggering duplication toxicity (Powers et al., 2023).

### 4.3 Next-generation vectors: TSHA-102 and miRNA-mediated autoregulation

To solve this, the field turned to nature’s own rheostat: microRNAs (miRNAs). The leading clinical candidate, TSHA-102 (developed by Taysha Gene Therapies), utilizes a novel “miRARE” (miRNA-responsive autoregulatory element) technology. This vector contains a “*mini-MECP2*” gene (optimized for size) coupled with a 3′UTR panel of binding sites for miRNAs that are highly expressed in the brain. In cells with high endogenous MeCP2 (healthy cells), these miRNAs bind the vector transcript and degrade it, preventing overexpression. In MeCP2-null cells, the transcript is translated freely because the expression of these specific regulatory miRNAs is positively correlated with MeCP2 levels. In the absence of MeCP2, the endogenous levels of these miRNAs are significantly reduced. Consequently, the RNA-induced silencing complex (RISC) is not efficiently recruited to the transgene’s 3′UTR, allowing the mRNA to evade degradation and undergo robust translation. Once MeCP2 protein levels are restored by the therapy, it upregulates these miRNAs, triggering a negative feedback loop that limits further transgene expression (Sinnett et al., 2021; Sadhu et al., 2023). This “smart” vector acts as a thermostat, maintaining protein levels within the safe physiological range. However, a theoretical concern remains regarding the “sponge effect,” where high levels of vector transcript might sequester endogenous miRNAs, potentially disrupting their normal regulatory functions (Moshiri et al., 2014). Long-term safety monitoring will be crucial to rule out such off-target perturbations.

### 4.4 Current clinical trials landscape (safety and efficacy data)

As of 2024, two landmark gene therapy trials are underway. TSHA-102 (REVEAL Trial, NCT05606614), an intrathecal AAV9 gene therapy using the miRARE platform, has shown preliminary Phase 1/2 data indicating the drug is generally well-tolerated. While long-term monitoring for potential overexpression toxicity remains a priority, early reports suggest improvements in autonomic function and communication, with no severe adverse events (SAEs) related to the vector reported to date (Percy et al., 2024). Concurrently, NGN-401 (Neurogene, NCT05898620) utilizes the “EXACT” technology to regulate transgene expression, with early safety data also appearing promising (Samanta, 2026). These trials represent the first attempts to treat the genetic root cause of RTT in humans, marking a definitive shift from symptomatic management to disease modification (Percy et al., 2024).

## 5 Therapeutic strategy II: epigenetic editing and reactivation

### 5.1 The holy grail: reactivating the silent X-chromosome (XCI)

Since females with RTT possess a healthy *MECP2* allele on the inactive X-chromosome, the most elegant cure would be to “wake it up.” This approach, known as X-Chromosome Inactivation (XCI) reactivation, theoretically avoids the risks of overexpression associated with exogenous gene transfer (Panayotis et al., 2023). Historically, global demethylating agents (e.g., 5-azacytidine) showed promise in cells but were too toxic for systemic use (Carrette et al., 2018). The field has struggled to find a way to selectively reactivate the inactive X without disrupting the entire epigenome.

### 5.2 Programmable epigenome editing (CRISPR-dCas9 systems)

A major breakthrough in XCI reactivation involves targeting the Xist interactome directly. Research has demonstrated that a mixed modality approach—combining specific small molecule inhibitors (such as PDPN) with antisense oligonucleotides (ASOs) targeting Xist—can synergistically disrupt Polycomb repressive complexes, leading to partial reactivation of the silent X-chromosome and amelioration of RTT phenotypes in mice (Carrette et al., 2018). However, global reactivation of the inactive X-chromosome poses a significant risk of “X-linked gene dosage imbalance,” where hundreds of genes usually silenced are overexpressed (Westervelt et al., 2021). Therefore, the field is prioritizing targeted epigenetic editing specifically directed at the *MECP2* promoter to avoid genome-wide toxicity. By fusing dead-Cas9 (dCas9) to demethylase domains (e.g., TET1), researchers can specifically strip methylation marks from the *MECP2* promoter on the inactive X, achieving “targeted reactivation” (Qian et al., 2023).

Crucially, regarding mechanism-to-treatment compatibility, targeted epigenetic reactivation stands out as a “mutation-agnostic” strategy. While pharmacological chaperones are limited to stabilizing misfolded proteins (e.g., T158M), and RNA editing strictly requires a targetable point mutation (e.g., G > A transitions), epigenome editing bypasses the underlying molecular dysregulation entirely. Whether the pathology stems from defective liquid-liquid phase separation (LLPS), a failure of NCoR/SMRT co-repressor recruitment, or the production of truncated nonsense proteins, activating the pristine wild-type MECP2 allele on the inactive X chromosome provides complete functional compensation (Tillotson and Bird, 2019). This broad compatibility positions epigenetic therapies as a universally applicable avenue for classic RTT patients, contrasting sharply with strictly genotype-dependent approaches. These “Epigenetic Rebooting” strategies represent the frontier of precision medicine.

### 5.3 RNA editing: correcting mutations at the transcript level

For patients with specific point mutations (e.g., G > A transitions), RNA editing offers a reversible and potentially safer alternative to DNA editing. By recruiting endogenous ADAR (adenosine deaminase acting on RNA) enzymes to the mutant *MECP2* transcript, specific adenosines can be converted to inosines (read as guanosine), effectively correcting the mutation at the RNA level (Sinnamon et al., 2020). This approach avoids permanent genomic changes and the risk of off-target DNA cleavage, presenting a promising avenue for “mutation-specific” therapy (Salvador et al., 2025).

### 5.4 DNA genome editing: direct correction of MECP2 mutations

While RNA editing provides a reversible approach, permanent correction at the DNA level represents the ultimate curative strategy for RTT. Traditional CRISPR/Cas9 systems rely on homology-directed repair (HDR) following double-strand breaks (DSBs) to correct genetic sequences. However, because mature neurons are post-mitotic, HDR is highly inefficient in the central nervous system, and the induction of DSBs carries a significant risk of causing unwanted insertions or deletions (indels) and cellular toxicity (Pickar-Oliver and Gersbach, 2019).

**FIGURE 3:**
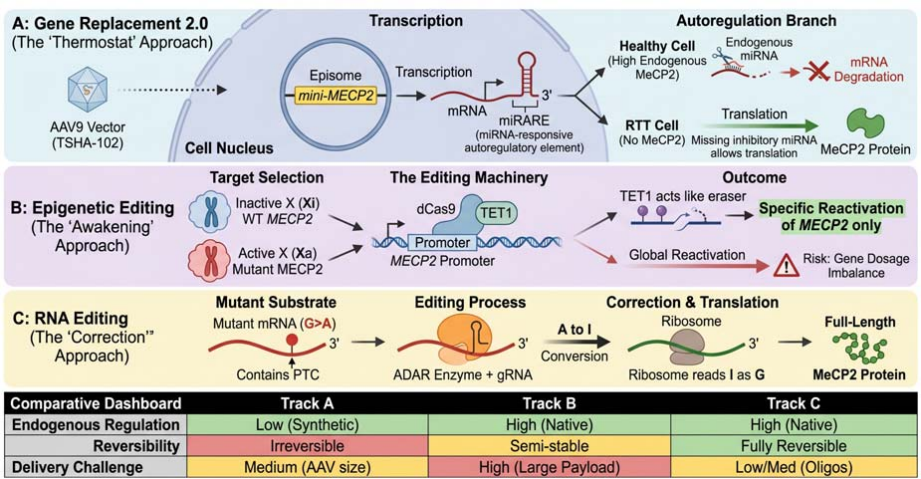
Mechanistic landscape of next-generation therapeutic strategies for Rett syndrome.

To circumvent these limitations, the field is rapidly shifting toward next-generation genome editing technologies, specifically base editing (BE) and prime editing (PE). Base editors can chemically convert one specific DNA base to another without requiring DSBs or donor DNA templates. This makes them exceptionally suited for correcting the precise point mutations (e.g., R168X, T158M) that account for the majority of RTT cases. Recent preclinical studies have demonstrated that CRISPR base editing can effectively correct specific *MECP2* variants, mapping mutational impacts and paving the way for *in vivo* functional rescue (Erwood et al., 2022). Furthermore, prime editing offers even broader versatility by rewriting short DNA sequences, which could potentially address small insertions and deletions in the *MECP2* gene. Despite this immense potential, translating these DNA-editing tools into clinical practice requires overcoming formidable delivery barriers, as the large physical size of BE and PE machineries currently exceeds the packaging capacity of standard viral vectors like AAVs. The main gene therapy and epigenetic editing strategies for restoring functional MECP2 are summarized and compared (Figure 3).

## 6 Therapeutic strategy III: downstream pathway modulation

### 6.1 Re-balancing the brain: pharmacological interventions

While gene therapies target the root cause, they may not be accessible to all patients immediately. Downstream pharmacological interventions remain vital. Trofinetide, approved by the FDA in 2023, represents the first success in this category. As a synthetic analog of the N-terminal tripeptide of IGF-1 (Glycine-Proline-Glutamate), it combats neuroinflammation and improves synaptic plasticity, providing modest but significant clinical benefits (Parent et al., 2023). However, its clinical utility is often limited by tolerability issues, particularly gastrointestinal distress (diarrhea) and weight loss, which frequently lead to dose reductions or discontinuation (Camillo et al., 2024). This highlights the need for more targeted therapies with better safety profiles. Another promising agent is Blarcamesine (Anavex 2-73), a Sigma-1 receptor agonist currently in Phase 3 trials. By activating Sigma-1 receptors, it enhances mitochondrial bioenergetics and reduces cellular stress, addressing the metabolic deficits inherent in RTT (Kaufmann et al., 2019).

### 6.2 BDNF mimetics and enhancers

Since BDNF deficiency is a core downstream pathology, restoring neurotrophic support is a logical strategy. However, BDNF itself has poor blood-brain barrier permeability. Small molecule TrkB agonists (e.g., LM22A-4) have shown efficacy in reversing respiratory deficits in mice by mimicking the BDNF loop domain (Schmid et al., 2012). Furthermore, low-dose ketamine has emerged as a potential modulator. At sub-anesthetic doses, it triggers rapid BDNF release and restores dendritic spine density without inducing the sedation or dissociative side effects associated with higher doses, offering a repurposing opportunity for this anesthetic as a synaptic rescuer (Kron et al., 2012).

### 6.3 Metabolic support: mitochondrial function and redox balance

Rett syndrome is increasingly viewed as a systemic metabolic disorder (Sette et al., 2026). MeCP2-deficient neurons exhibit defective mitochondrial respiration and increased oxidative stress (Müller, 2019; Woeste et al., 2025). Therapeutic strategies focusing on redox balance, such as mitochondrial antioxidants or metabolic co-factors, may serve as valuable adjuncts to genetic therapies, ensuring that revitalized neurons have the energy substrate required for plasticity (Enns et al., 2017).

## 7 Critical appraisal and future directions

### 7.1 The delivery barrier: crossing the blood-brain barrier (BBB) with large payloads

Despite the elegance of molecular designs, delivery remains the “Achilles’ heel” of genetic therapies. AAV9 vectors can cross the blood-brain barrier (BBB), but efficiency is limited. To achieve therapeutic levels in the brain, high systemic doses are required, which often lead to hepatotoxicity and dorsal root ganglion (DRG) pathology (Hordeaux et al., 2020; Aeran et al., 2025). The field is urgently exploring Next-Generation Capsids (e.g., engineered AAV variants) that are evolved specifically for enhanced CNS tropism and de-targeted from the liver (Deverman et al., 2016). Furthermore, for CRISPR-based epigenetic editing, the delivery challenge is compounded by the large size of dCas9 fusion proteins, which often exceed the packaging capacity of a single AAV (∼4.7 kb). This necessitates the development of dual-vector systems or non-viral delivery methods like lipid nanoparticles (LNPs) to transport the editing machinery effectively (Yang, 2025; Zhang et al., 2026).

### 7.2 Immunogenicity: managing the host response to viral vectors

The human immune system poses a formidable barrier. Many patients have pre-existing neutralizing antibodies to AAV9, rendering them ineligible for trials. Furthermore, the introduction of a “foreign” protein (MeCP2) or Cas9 enzymes can trigger a cytotoxic T-cell response, potentially eliminating the corrected cells (Serpa et al., 2025). Current protocols employ rigorous prophylactic immunosuppression (e.g., rituximab, sirolimus), but developing “stealth” vectors or tolerogenic strategies is critical for long-term efficacy (Potter et al., 2024).

### 7.3 Biomarkers: the quest for objective outcome measures

The approval of Trofinetide relied on caregiver-reported scales (RSBQ, CGI-I), which are subject to placebo effects and inter-rater variability, highlighting the need for objective, quantifiable biomarkers in precision medicine trials. Quantitative EEG (qEEG) offers one such avenue; recent studies have identified specific spectral signatures, particularly increased delta power (1–4 Hz) in the frontal regions, as robust correlates of RTT severity (Goodspeed et al., 2023). Normalization of these EEG patterns could serve as an early readout of synaptic restoration. Additionally, Fluid Biomarkers involving circulating microRNAs or metabolites that reflect brain health are currently being validated (He et al., 2025). Establishing these “surrogate endpoints” is essential to accelerate clinical trial timelines and reduce the burden on patients.

### 7.4 Ethical considerations in pediatric genetic interventions

Treating pediatric patients with irreversible genetic tools raises profound ethical questions (Kohn et al., 2020). The concept of “identity” is central—does restoring MeCP2 function change the person? Moreover, the high cost of gene therapies creates potential disparities in access (Garrison et al., 2019). A rigorous ethical framework must accompany technological progress to ensure that the benefits of precision medicine are equitably distributed (Knoppers et al., 2017).

### 7.5 Combinatorial approaches: “Cocktail” epigenetic therapies

The future of RTT treatment likely lies not in a “magic bullet,” but in combinatorial strategies (Katz et al., 2016). A patient might receive a gene replacement therapy to restore basal MeCP2 levels, coupled with a downstream BDNF enhancer to accelerate synaptic plasticity, and a metabolic modulator to support mitochondrial health. Such “cocktail” approaches could maximize functional recovery (Downs et al., 2024).

## 8 Conclusion

Rett syndrome has undergone a profound conceptual transformation, evolving from a static, irreversible condition defined solely by its clinical symptoms to a disorder characterized by its remarkable molecular reversibility. This paradigm shift, ignited by early proof-of-concept studies in animal models, has now matured into a robust clinical pipeline. The realization that the RTT brain is not structurally damaged but functionally dormant has provided the fundamental scientific rationale for pursuing curative rather than merely palliative interventions.

The therapeutic landscape is now populated by a diverse array of precision genetic medicines. From next-generation gene replacement therapies utilizing miRNA-mediated autoregulation to prevent toxicity, to the cutting-edge frontier of programmable epigenetic editing and targeted X-chromosome reactivation, RTT stands at the vanguard of neuro-epigenetic therapeutics. These strategies do not merely aim to alleviate symptoms but seek to correct the root cause of the disease. Moreover, the innovations pioneered here—such as “smart” vectors and fine-tuned epigenetic modulators—will serve as a crucial blueprint for treating a host of other monogenic neurodevelopmental disorders.

As we move from bench to bedside, the path forward requires a synergistic integration of deep mechanistic understanding with practical clinical solutions. Overcoming the remaining hurdles of delivery efficiency, immunogenicity, and the validation of objective biomarkers will be the key to unlocking the full potential of the neuronal epigenome. The concept of a “cure”—once a distant and ambitious dream—has now graduated into a tangible scientific hypothesis being rigorously tested in clinics, offering renewed hope to patients and families worldwide.
